# Asialoglycoprotein receptor targeted optical and magnetic resonance imaging and therapy of liver fibrosis using pullulan stabilized multi-functional iron oxide nanoprobe

**DOI:** 10.1038/s41598-021-97808-0

**Published:** 2021-09-15

**Authors:** Ariya Saraswathy, Shaiju S. Nazeer, Nirmala Nimi, Hema Santhakumar, Parvathy Radhakrishnapillai Suma, Kunnumpurathu Jibin, Marina Victor, Francis Boniface Fernandez, Sabareeswaran Arumugam, Sachin J. Shenoy, P. R. Harikrishna Varma, Ramapurath S. Jayasree

**Affiliations:** 1 Division of Biophotonics and Imaging, Biomedical Technology Wing, Sree Chitra Tirunal Institute for Medical Sciences and Technology, Poojappura, Thiruvananthapuram, Kerala 695 012 India; 2 Department of Physics, HHMSPBNSS College, Thiruvananthapuram, Kerala 695 040 India; 3 Department of Chemistry, Indian Institute of Space Sciences and Technology, Thiruvananthapuram, Kerala 695547 India; 4 Division of Bioceramics Laboratory, Biomedical Technology Wing, Sree Chitra Tirunal Institute for Medical Sciences and Technology, Poojappura, Thiruvananthapuram, Kerala 695 012 India; 5 Division of Implant Biology, Biomedical Technology Wing, Sree Chitra Tirunal Institute for Medical Sciences and Technology, Poojappura, Thiruvananthapuram, Kerala 695 012 India; 6 Division of In Vivo Models and Testing, Biomedical Technology Wing, Sree Chitra Tirunal Institute for Medical Sciences and Technology, Poojappura, Thiruvananthapuram, Kerala 695 012 India

**Keywords:** Biomaterials, Nanoscale materials, Materials science, Nanoscience and technology

## Abstract

Early diagnosis and therapy of liver fibrosis is of utmost importance, especially considering the increased incidence of alcoholic and non-alcoholic liver syndromes. In this work, a systematic study is reported to develop a dual function and biocompatible nanoprobe for liver specific diagnostic and therapeutic applications. A polysaccharide polymer, pullulan stabilized iron oxide nanoparticle (P-SPIONs) enabled high liver specificity via asialogycoprotein receptor mediation. Longitudinal and transverse magnetic relaxation rates of 2.15 and 146.91 mM^−1^ s^−1^ respectively and a size of 12 nm, confirmed the T_2_ weighted magnetic resonance imaging (MRI) efficacy of P-SPIONs. A current of 400A on 5 mg/ml of P-SPIONs raised the temperature above 50 °C, to facilitate effective hyperthermia. Finally, a NIR dye conjugation facilitated targeted dual imaging in liver fibrosis models, in vivo, with favourable histopathological results and recommends its use in early stage diagnosis using MRI and optical imaging, and subsequent therapy using hyperthermia.

## Introduction

Liver cancer is the third most leading cause of cancer death worldwide^1^. Hepatitis virus infection and alcohol abuse are the major cause behind the high incidence of this disease. Hepatic architectural damage is the prime feature of most of the chronic liver diseases. Fatty liver is the earliest stage of liver damage which develops through fibrosis to cirrhosis with a high chance of transforming it to hepatocellular cancer (HCC)^2,3^. An efficient management of the disease is possible if diagnosed at the curable stage of liver fibrosis whereas the management becomes difficult or rather not possible on advancement of the condition to cirrhosis and HCC.

Various in vivo imaging modalities like Computed Tomography, MRI, Ultrasound and Positron Emission Tomography have been used for cancer diagnosis. Among them MRI provides better sensitivity and specificity with high spatial and temporal resolution. Even though MRI provides images with intrinsic contrast, authentic diagnosis of diseases like liver associated malignancies often requires the use of external agents to enhance the image contrast for better visualization. For the diagnosis of early fatty changes within the liver, signal from the transverse relaxation of protons (T_2_) is preferred over longitudinal relaxation (T_1_). This is due to low T_1_ response of accumulated fat within liver than hepatocytes^4^. Due to this reason, contrast agents with enhanced transverse relaxivity such as surface modified super paramagnetic iron oxide nanoparticles (SPIONs) are preferred for liver imaging over conventional gadolinium-based contrast agents with longitudinal relaxation properties.

Application of iron oxide based transverse contrast agents includes liver, lymph node and vascular MR imaging^5–7^. Non-specificity is one of the major drawbacks of these blood pool contrast agents. Most of the iron oxide based T_2_contrast materials have been withdrawn from the market due to safety concerns^8^. It is reported that low molecular weight polymer coatings have a tendency to cause osmotic nephropathy that leads to chronic renal failure^7,9^.

Surface modification of iron oxide nanoparticles with biocompatible, water soluble polymers of high molecular weight is an efficient way to overcome the safety concerns of currently available iron oxide based MRI contrast agents. Hence, a polysaccharide, pullulan, the malto-triose units of which has high specificity towards ASGPR over expressed on sinusoidal membranes of hepatocytes is used as the coating agent, in this study^10^. Pullulan is a water-soluble non-ionic exopolysaccharide of fungal origin containing repeated malto-triose units condensed through α-1,6 linkage. Pullulan has been found suitable for various biomedical applications including tissue engineering, imaging, targeted drug and gene delivery. It also has improved biocompatibility, biodegradability and transfection efficiency, which makes it suitable for various biomedical applications^11–14^.

In this study, hepatocyte-specific pullulan modified SPIONs with hydrodynamic size of 80 nm were synthesized for theranostic application of liver diseases. The synthesized P-SPIONs showed excellent magnetic relaxivity enabling high contrast for MRI. The heat generation capacity of P-SPIONs under the influence of alternating current favoured magnetic hyperthermia based therapeutic intervention. Moreover, P-SPIONs were further conjugated with an NIR emitting dye Atto-700 (PSPION-AT) to impart dual imaging capability for the material. P-SPIONs also showed good blood and cell compatibility even at higher concentrations. Efficacy of the developed system for the hepatocellular targeting and early diagnosis of liver fibrosis was demonstrated in rodent model of liver fibrosis. A comparative evaluation of the hepatocellular uptake efficiency and contrast enhancement of P-SPIONs with citrate and dextran coated SPIONs have also been carried out^15,16^.

## Materials and methods

### Materials

Pullulan (Mw ~ 100,000) (Sigma Aldrich, St. Louis, MO, USA), FeCl_3_ anhydrous, FeCl_2_.4H_2_O, NaOH, and 35% HCl (Merck, Germany/India) were used for the preparation of iron oxide nanoparticles as reported in our earlier works^15–17^. The chemicals used for the cell culture studies were 3-[4,5-dimethylthiazol-2yl]-2,5-diphenyltetrazolium bromide (MTT), F12K medium, sodium bicarbonate, gentamicin (Himedia, India), amphotericin B solution and fetal bovine serum (FBS) (Sigma-Aldrich, Germany). Carbon tetrachloride (CCl_4_) and olive oil were used for the development of animal model for liver fibrosis. All the chemicals were used as received without any further purification. Water used in all experiments was purified using a Milli-Q Plus185 water purification system with resistivity higher than 18 MU cm.

### Synthesis of P-SPIONs

For the synthesis of SPIONs, we have followed a standardized synthesis method as reported earlier^15,16^. Briefly, a solution consisting of FeCl_3_ and FeCl_2_ with a molar ratio of 2:1 was mixed under N_2_ protection, followed by gradual addition of 1 M NaOH under vigorous stirring to obtain black Fe_3_O_4_ precipitate (SPIONs). The reaction continued for 2 h to ensure absolute precipitation. Then, the obtained SPIONs were separated magnetically and washed immediately with distilled water. The above prepared SPIONs were re-dispersed in 1.2% pullulan to make the nanoparticles more stable, biocompatible and hepatocyte specific. The solution mixture was stirred for 12 h. Magnetic separation was carried out to remove excess pullulan. After washing several times, P-SPIONs was dispersed in 10 mL deionised water and used for the rest of the studies. For the preparation of PSPION-AT, 20 μL of 1.3 μg/μL concentrated Atto dye was added to 10 ml of 1.2 mg/mL P-SPIONs and ultra-sonicated for 6 h. The final suspension was centrifuged and used for fluorescent studies.

### Physico-chemical characterization

The core size of P-SPIONs was determined using transmission electron microscopy at 100 kV (TEM, JEM-2010, Hitachi-JEOL, Tokyo, Japan). The hydrodynamic diameter and zeta potential were measured using dynamic light scattering method with Malvern Zetasizer nano ZS apparatus (Malvern Instruments, Malvern, UK). Using X’Pert PRO X-ray diffraction (XRD), phase and crystalline properties were investigated. Crystal structure was determined from the position and intensities of diffraction peaks observed in the diffraction angle range 2θ = 10–80° using Cu Kα radiation of 1.54 Å at 40 kV and 20 mA current. The conjugation of SPIONs to pullulan was confirmed via Fourier transform infrared spectroscopy (FTIR) on a Thermo Nicolet 5700 FTIR spectrometer (USA). Spectra were recorded as KBr pellets over the range 4000–400 cm^−1^ at spectral resolution of 4 cm^−1^.Thermogravimetric analysis (TGA) was used to evaluate the thermal decomposition properties of the prepared materials. TGA was also used to evaluate the amount of pullulan bound to the SPIONs. TGA of lyophilized P-SPIONs and pullulan were performed using SDT 2960 V2.2B (Simultaneous TGA-DTA, TA Instruments, Delaware, USA). TGA was run with in a temperature range of 25–1200 °C applying a constant heating rate of 10 °C/min.

### Magnetic property of P-SPIONs

Magnetic properties of solid P-SPIONs was measured with a Lakeshore model 7410 vibrating sample magnetometer (VSM) using maximum fields of 150 Oe. The saturation magnetization (Ms) was determined from M versus H plots and extrapolated to infinite fields. For magnetic relaxivity measurements, homogeneous solutions of different concentrations of phantoms ranging from 0 to 0.45 mM Fe were scanned using a 12 channel RF coil of 1.5 Tesla whole body MRI scanner (MAGNETOM Avento Tim, Siemens, Munich, Germany). Longitudinal relaxation time, T_1_ of the samples was measured using an inversion recovery MRI sequence. The repetition time (TR) and echo time (TE) were set at 4000 and 11 ms respectively and the MR signal was measured by changing the inversion time (TI) from 50 to 3000 ms. T_2_ relaxometric measurements were run with a modified T_2_ relaxometry spin echo sequence. For a fixed TR of 2000 ms, MR signal was measured for varying TE of 15 to 120 ms. T_1_ and T_2_ relaxation times were calculated from the resulting MRI pixel intensity maps with respect to each concentration. The relaxation rates, r_1_ and r_2_ values were calculated and plotted against the iron concentration, and relaxivity value was determined by the linear regression^17–20^.

### Magnetic hyperthermia

The heat generation capacity of magnetic nanoparticles (P-SPIONs) was investigated using Ambell EASY HEAT laboratory induction system with a magnetic field frequency of 275 kHz. A solenoid coil with a 4 cm diameter, 2.6 cm length and a total of 6 turns was set as the sample compartment. The sample was subjected to an alternating magnetic field (AMF). The temperature of the nanoparticle suspension was measured as a function of time. The current was changed from 200 to 400 A in steps of 50 A at a fixed frequency of 275 kHz. The induction heating treatment was performed by suspending the sample particles in 1 ml of distilled water in a 1.5 ml non-magnetic vial kept at the center of the coil. The temperature profile was recorded for 15 min using noncontact mode IR thermometer (Fluke 572, Germany). From this temperature profile result, specific loss power (SLP) of developed SPIONs and P-SPIONs were evaluated using the following equation^21^.

$$SLP = \frac{CVs}{m}\frac{dT}{{dt}}$$

where C is the volumetric specific heat capacity of the sample (C_water_ = 4185 J L^−1^ K^−1^ C), Vs is the sample volume, m is the mass of the magnetic material present in the sample volume and dT/dt is the initial linear rise in temperature versus time dependence^21^.

### Hemocompatibility

Blood aggregation studies and haemolysis assay of P-SPIONs were evaluated on red blood cells (RBC), white blood cells (WBC) and platelets, as per the reported protocols^22–24^. Saline was used as negative control and polyethyleneimine (PEI) as positive control. Aggregation was assessed by checking the morphology of the cells using phase contrast microscopy (Leica DM IRB, Germany). For haemolysis assay, the acquisition of absorbance spectra in the range 400 to 600 nm was carried out and absorbance at 541 nm was measured on the supernatants of RBCs incubated with samples of different concentration.

### In vitro evaluations

#### In vitro cytotoxicity

Cytotoxicity of P-SPIONs on human hepatocellular carcinoma cells, Hep G2, and mouse fibroblast cells, L-929 was assessed by standard MTT assay, in triplicate^15,16,25^. Different concentrations of P-SPIONs (100,50 and 25 µg/mL), negative and positive controls were added to the cells and incubated for 24 h. On removal of the media, MTT (0.2 mg/mL) was added to each well and again incubated for 3 h. On removal of MTT, dimethyl sulfoxide was added to dissolve the formed formazan crystals and incubated for 30 min. Absorbance was quantified based on the peak at 570 nm.

#### Reactive oxygen species (ROS) quantitative measurement

Reactive oxygen species detection reagent, 2′7′-dichlorodihydrofluorescein diacetate, acetyl ester (DCFDA) was used to detect ROS levels in Hep G2 cells upon addition of P-SPIONs^26^. The cells were seeded on a 96 well plate at a density of 1 × 10^4^ cells/well and allowed to grow overnight. A day later, the cells were incubated with P-SPIONs for 24 h. 10 μM H_2_O_2_ was made use as a positive control for the assay. After 24 h, the treatment medium from each well was replaced with 100µL of fresh DMEM without phenol red indicator and serum, containing 10 mM DCFDA and allowed to incubate in dark for 20 min at 37 °C temperature. The fluorescence intensity of the formed 2’, 7’-dichlorofluorescein as a result of carboxy-DCFDA hydrolysis, was analyzed in a 96well spectrofluorometer plate reader at an excitation and emission wavelength of 492 and 527 nm respectively.

#### Cell uptake study

HepG2 cells were incubated with P-SPIONs and the uptake and localization of particles in the cells were confirmed using iron specific Prussian blue staining.

#### In vitro magnetic hyperthermia

HepG2 cells were cultured in DMEM medium (Himedia Laboratories Pvt Ltd, Mumbai, India) supplemented with 10% fetal bovine serum (FBS, Himedia) and an antibiotic-antimitotic mix (Himedia) at 37 °C in a humidified and 5% CO_2_ atmosphere. For the in vitro magnetic hyperthermia treatment (MHT) experiments, cells were seeded onto 12-mm cover slips with a seeding density of 10,000 cells kept inside a 24-well culture plate. After 24 h of incubation, cell media was replaced with fresh DMEM media containing 2 mg/mL of sterile P-SPIONs and the cells were incubated at 37 °C. The P-SPIONs incubated cells were subjected to magnetic hyperthermia using an alternating magnetic field of 33.8 mT and 275 kHz applied for 15 min. The temperature change of the cell environment was monitored using an infrared thermometer. The cell viability after magnetic hyperthermia treatment were studied by live/dead assay using acridine orange and ethidium bromide stains and observed under Olympus IX83 inverted fluorescence microscope. Cells without any treatment served as control and those incubated with 2 mg/mL P-SPIONs without AMF treatment were used for the comparison. The morphological features of the treated cells were studied using SEM. Cells treated with magnetic hyperthermia were washed with sterile PBS and fixed with 2% glutaraldehyde in PBS overnight at 40 °C. The samples were washed thrice with PBS and dehydrated through a series of alcohol concentrations (30%, 50%, 70%, 90% and100%) and subjected to critical-point drying. The samples were then coated with gold and examined by environmental scanning electron microscopy (FEI QUANTA 200)^27^.

### Optical property

The optical properties of the PSPION-AT were characterized by measuring absorbance and fluorescence. Optical imaging system (Xenogen IVIS in vivo imaging system) also was used to test the imaging efficiency. The detailed information about the excitation, emission and contour plots of PSPION-AT is given in Figs. S1 and S2.

### In vivo MR imaging

All experimental protocols involved in animal experiments were approved by the Institutional Animal Ethics Committee of Sree Chitra Tirunal Institute for Medical Sciences and Technology (Order no: B 2982011 IX, dated: 19-10-2011) in accordance with the regulations of Committee for the Purpose of Control and Supervision of Experiments on Animals, India. All methods were performed in accordance with the relevant guidelines and regulations. Animal study was carried out in compliance with the ARRIVE guidelines.

#### Animal model for liver fibrosis

Male Wistar rats (n = 6) with an average body weight of 220 g were used for the development of hepatic fibrosis model as per reported protocol^28^. Briefly, CCl_4_ was dissolved in olive oil (1:1), and 1µL/g body weight of the mixture was injected intra-peritoneally twice a week for 6 weeks. Liver function tests (LFT) for the enzymes, aspartate aminotransferase (AST) and alanine transaminase (ALT) confirmed the development of fibrosis. For optical imaging, liver fibrosis was developed in male Swiss albino mice (n = 6) by treating 1:7 ratio of CCl_4_:olive oil mixture intra-peritoneally at the dosage of 1 μL/g body weight, every 5 days, for 4 weeks. Liver function tests and post-mortem histopathology confirmed fibrosis development.

#### In vivo MRI

MRI was performed on a 1.5 T MR scanner (Siemens, Germany) using a head coil. Multi-section T_2_-weighted TSE sequence with the parameters TR 5780 ms; TE 125 ms; FOV 98 X 140 mm; slice thickness 3 mm; flip angle 90 were set for imaging. The animals were anaesthetized using ketamine and xylazine mixture at a dosage of 70 mg/kg and 5 mg/kg of body weight prior to MRI scan. Both pre and post-contrast T_2_ weighted images were acquired without disturbing the set parameters. After acquiring pre-contrast images, P-SPION was injected through tail vein at a dose of 2.17 mg/ml (0.04 mM) of Fe/kg bodyweight and post-contrast images were acquired within 10–30 min. Pre and post contrast images were quantified for pixel intensity using the Leonardo workstation (Siemens, Germany).

#### In vivo optical imaging

In vivo optical imaging of mice was performed using the Xenogen IVIS spectrum in vivo optical imaging system having excitation filters in the range 430–780 nm and emission filters in the range 500–800 nm^29,30^. A series of images are acquired with different combinations of excitations and emissions in the above range. The control and test animals are imaged side by side so that the difference between the two will be observed as the net fluorescence signal. P-SPION-AT was injected through tail vein at a dose 0.04 mM of Fe/kg bodyweight for optical imaging of fibrosis. Saline injected fibrosis induced animals served as control.

### Histopathology

On sacrificing the animals after 2 h of P-SPIONs injection and MR imaging, liver was extracted and fixed with paraformaldehyde and embedded in paraffin for histological analysis. Haematoxylin and eosin staining (H&E) was performed according to standard protocols. The special stains, Masson’s Trichrome (MT) for the detection of collagen fibres and the Pearls’ Prussian blue (PB) for assessing the presence of iron was also carried out.

## Results and discussion

### Synthesis of P-SPIONs

**Figure 1:**
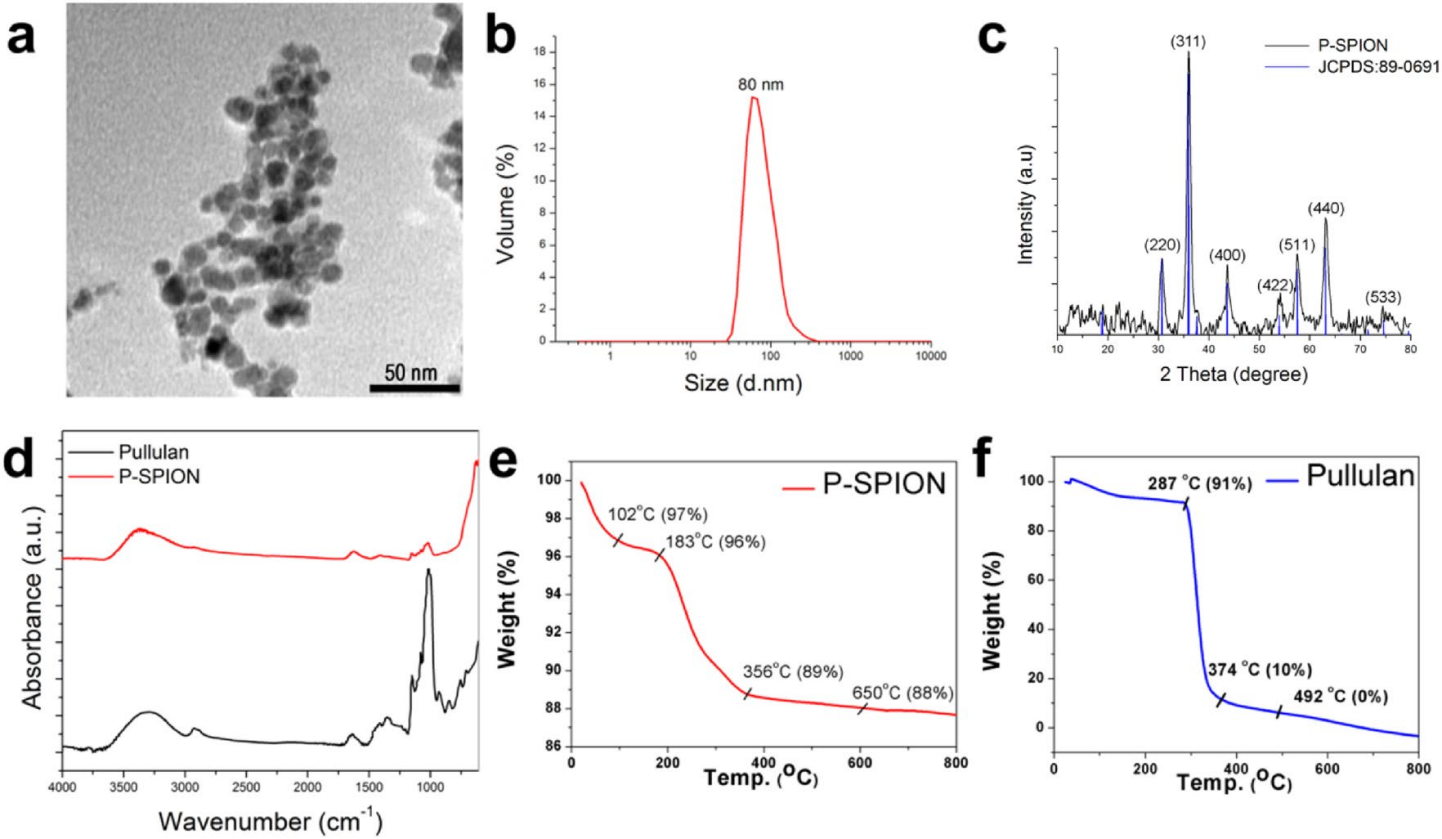
Physico-chemical characterization of P-SPIONs. (**a**) TEM and (**b**) DLS graph of P-SPIONs, (**c**) and (**d**) represent its XRD pattern and FTIR spectra respectively. (**e**) and (**f**) indicates the TGA curves of P-SPIONs and Pullulan.

A simple synthesis route was adopted for the formulation of P-SPIONs to develop a hepatocyte-specific MR contrast and hyperthermia agent. As is clear from TEM and DLS measurements, the core size and hydrodynamic diameter of the prepared P-SPIONs were 12 ± 2 nm and 80 nm respectively without any aggregation (Fig. 1a,b) compared to bare SPIONs (Fig. S3). The XRD pattern shows intense diffraction peaks at 2θ = 30.1°, 36.2°, 42.4°, 57.2° and 62.8° due to face centered cubic lattice structure with the corresponding indices (220), (311), (400), (440) and (511) which are characteristic of magnetite (Fe_3_O_4_) crystal (Fig. 1c). The weak peak corresponding to that of γ-Fe_2_O_3_ at 31° for the indices (210) and (213) suggests the presence of maghamite traces in the developed nanosystem^31^. Surface modification of Fe_3_O_4_ with pullulan was confirmed by FTIR spectroscopic analysis (Fig. 1d) and thermal analysis (Fig. 1e–f). The pullulan stabilized SPIONs also exhibited the characteristic peaks of pullulan with diminutive shifts in addition to the parent SPION formulation. The peaks are assigned as 3368 (O–H stretch), 2924 (–CH2 stretching vibrations), 1623 (C–O stretching), 1409 (C–H bending), 1018 (C–CO stretching) and 930 cm^-1^ (–CH out-of-plane bending) respectively (Fig. 1d). FT-IR spectrum of bare SPION is shown in the supporting information as Fig. S4. From the TGA curve of pure pullulan (Fig. 1f), the rapid thermal decomposition is observed at 250 °C with a complete dissociation at 625 °C. This agrees with observations reported by Karim and team^32^. The thermal degradation of pullulan in P-SPIONs was shifted to 183 °C with a weight loss of 4% and the complete dissociation was observed after 625 °C with a total weight loss (12%) (Fig. 1e).The shift in the degradation peaks of pullulan in the coated system corresponds to catalytic behavior of the iron oxide core. Furthermore, the zeta potential measurement of P-SPIONs confirm the pullulan coating over iron oxide nanoparticles with an increase in the negative surface charge potential of − 14.6 mV from − 9.29 mV of SPIONs.

### Magnetic property and hyperthermia

**Figure 2:**
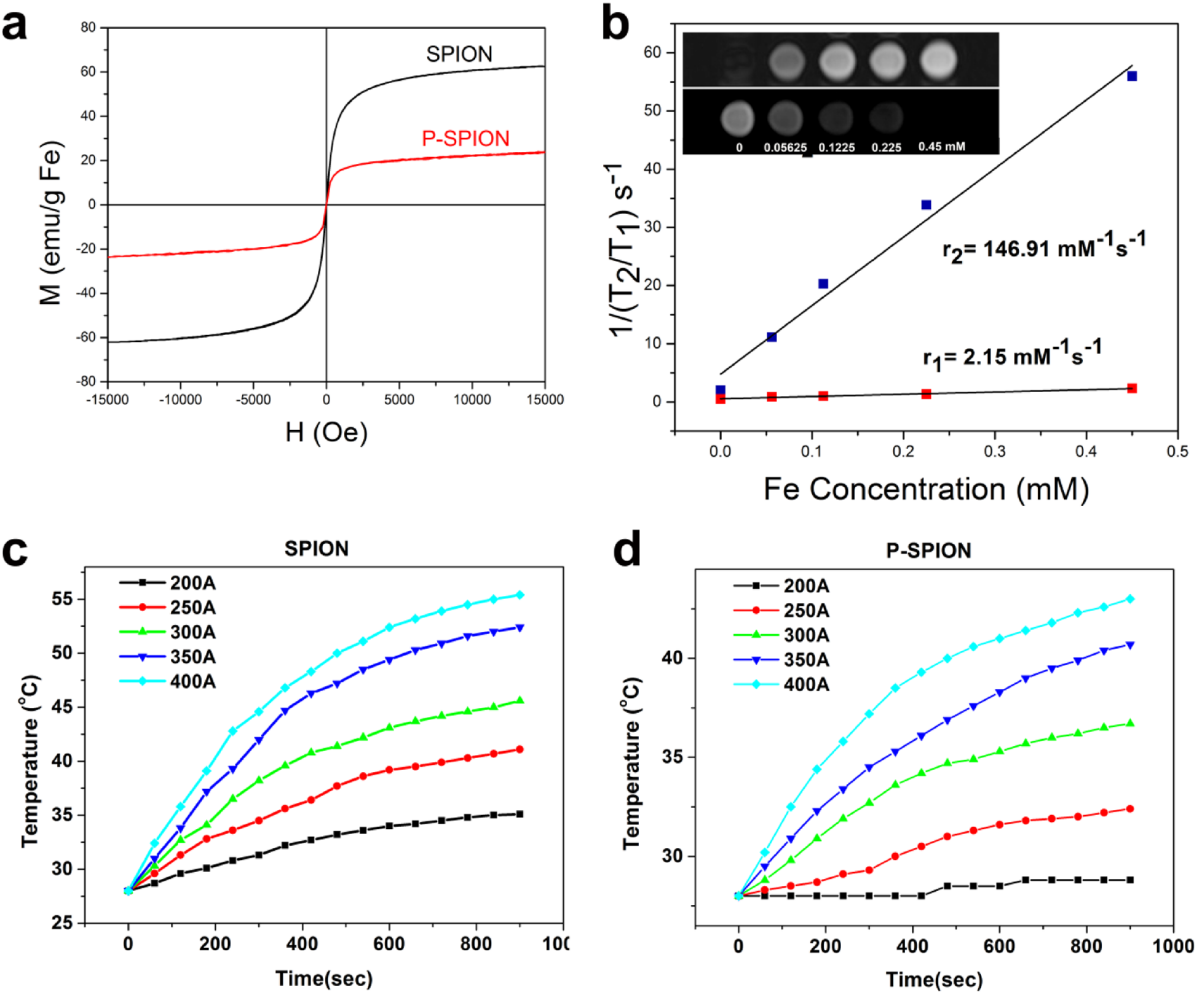
Magnetic and hyperthermic effects of P-SPIONs. (**a**) and (**b**) represents the super paramagnetic property and relaxivity effects of SPIONs and P-SPIONs respectively (inset represents the T_1_ (Upper) and T_2_ (lower) contrast MRI images of P-SPIONs). (**c**) and (**d**) represents the hyperthermia effects of SPIONs and P-SPIONs respectively.

The hysteresis curves of both P-SPIONs and bare SPIONs showed neither remanence nor coercivity, indicating the super-paramagnetic nature, and these were completely reversible at 300 K (Fig. 2a). Superparamagnetic property of the nanoparticles is important for using them as T_2_ contrast agents in MRI. A reduction in the saturation magnetization of P-SPION is observed compared to uncoated SPION. It has been reported that the magnetic phase in nanoparticles varies with different coatings as well as with solvents^33–35^. The reason for magnetic phase reduction has been explained as due to the surface spin disorder influenced by the absorbance of coatings and electric charge. It is also attributed to the effect of the solvents, which may change the strength of the interaction of the coating and the magnetic core to form a nonmagnetic layer. Since in this study the solvent used is the same, the reduction in magnetic property is attributed to the spin disorder due to the extra layer of non magnetic material over the core. Pullulan, a high molecular weight polymer, may interact with the surface atoms of the magnetic core and form a magnetically disordered layer, reducing the total amount of the magnetic phase and is responsible for the observed reduction in the magnetic property. However, the magnetic property exhibited after the coating was found to be good enough for theranostic application.

The relaxation effects of P-SPIONs were evaluated using a 1.5 T MR scanner. On the T_2_ weighted images, the pixel intensities decrease with increase in concentration of Fe (0 to 0.45 mM) in the aqueous phantoms. Compared to the control, increase in the P-SPIONs concentration resulted in the effective increase in the hypo intense contrast (Fig. 2b inset). The T_1_ weighted images of phantoms exhibited brighter contrast with the increase in concentration of P-SPIONs. The longitudinal and transverse relaxation rates (r_1_ & r_2_) were calculated from the pixel intensities of respective phantom images (Fig. 2b). From the plot of relaxation rate versus Fe concentrations, the r_1_ and r_2_ values were calculated as 2.15 and 146.91 mM^−1^ s^−1^ with r_2_/r_1_ ratio of 68.33. The r_2_/r_1_ ratio, which is an indicator of relaxometric properties of contrast agent, classifies P-SPIONs as an efficient T_2_ contrast agent^4,36^.

In addition, P-SPIONs samples were tested for its potential for hyperthermia as compared to SPIONs and results are presented in Fig. 2c,d. A concentration of 5 mg/mL raised the temperature to more than 50 °C on the application of 400A current for 8 min, proving its candidature for hyperthermia. However, it is noted that effective hyperthermia using lower concentrations of the order of 1 mg/mL has been reported, earlier^37^.

Evaluation of specific loss of power (SLP) of a magnetic material can act as clear indicator for the material’s therapeutic efficacy. SLP is defined as the amount of energy converted into heat per time and mass. Theraputic capability of SPIONs and P-SPIONs were determined on the basis of SLP. The calculated SLP values are given in Table S1. It is observed that on increasing the field strength, developed magnetic nanoparticles produced elevated temperature and saturations. As expected the SLP of P-SPIONs are observed to be less than that of SPIONs, due the surface modification using pullulan and subsequent reduction in the magnetic property^38^.

### In vitro evaluations

**Figure 3:**
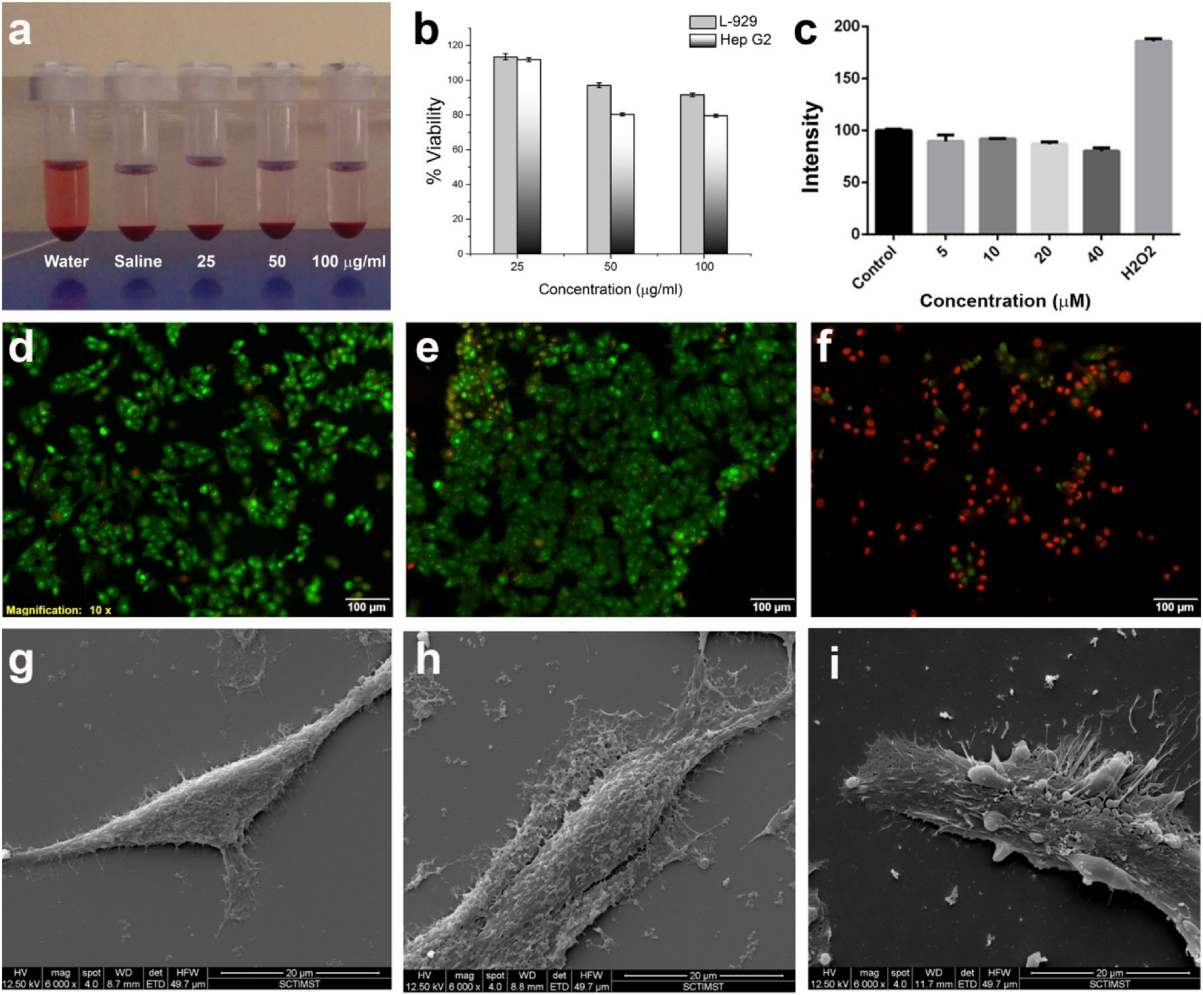
In vitro evaluation of P-SPIONs. (**a**) Hemocompatibility assay and (**b**) cell viability assay of P-SPIONs. (**c**) Represent the quantification of ROS generated from P-SPIONs through DCFDA assay. (**d**–**f**) Live/dead assay and (**g**–**i**) ESEM images of HepG2 cells subjected to hyperthermia treatment of P-SPIONs.

Hemocompatibility, a primary requirement for in vivo application, was evaluated by the hemolysis assay and the blood aggregation studies^24^. Non hemolytic nature was clear for different concentrations of P-SPIONs (Fig. 3a). Saline and water were used as negative and positive controls respectively. The percentage of lysis was found to be less than 1% for different concentrations (25 μg/ml—0.32%, 50 μg/ml—0.55%, 100 μg/ml—0.71%) of P-SPIONs. No aggregation in any of the cells confirms the blood compatibility of the developed particles (Fig. S5). Then, the cytotoxicity of P-SPIONs was evaluated using human liver carcinoma cell, HepG2 and Normal fibroblast cells L-929 using MTT assay. A dose-dependent response was observed at 24 h, in cells treated with P-SPIONs (concentrations chosen were in the range 25–100 µg/mL). More than 90% of the cells were viable at concentration as high as 100 µg/ml of P-SPIONs, indicating the cyto-compatibility (Fig. 3b).

The intracellular ROS created by P-SPIONs was evaluated through 2′,7′-dichloro fluorescindiacetate (DCFDA) analysis. Even at the high concentrations of 5 µM to 40 µM, the fluorescence signals remained almost the same for P-SPIONs treated HepG2 cells which is on a par with the fluorescence signals from the control cells, at 24-h post-treatment (Fig. 3c). After subtracting the basal level of reactive oxygen from all the treatment concentrations, there is no observable hike in the fluorescence signals even at the highest treatment concentration. In fact, there is an insignificant reduction in the fluorescence intensity which might be due to the scavenging of ROS by the cells by its inherent mechanism to overcome any adverse cell activity in the presence of a foreign body.

The cellular uptake and labeling efficiency of P-SPIONs in HepG2 cells are shown in Fig. S6 where iron specific PB staining is performed and is compared with the control cells without any particles. The image gives an indication of the effective uptake of the materials by the cells without any targeting molecules due to the high density of ASGPR receptors present on these cells^39^. The particles are mostly concentrated in the cytoplasm. The normal morphology of the cells remained unaltered after 24 h of incubation, indicating the cell compatibility of P-SPIONs.

The suitability of the developed material for hyperthermia was also evaluated, in a biological environment. For this, P-SPIONs incubated with HepG2 cells were exposed to alternative magnetic field (AMF) for 15 min. Majority of the cells after AMF exposure were positively stained for cell death compared to the control cells and cells with P-SPIONs and without AMF exposure (Fig. 3d–f). Control cells with acridine orange vital stain showed the characteristic green signal of live cells. ESEM images of the cells treated with P-SPIONs and AMF showed cell membrane rupture, loss of characteristic cell morphology and membrane blebbing indicating the induction of apoptosis which leads to cell death (Fig. 3g–i).

### In vivo MRI of liver fibrosis animal model

**Figure 4:**
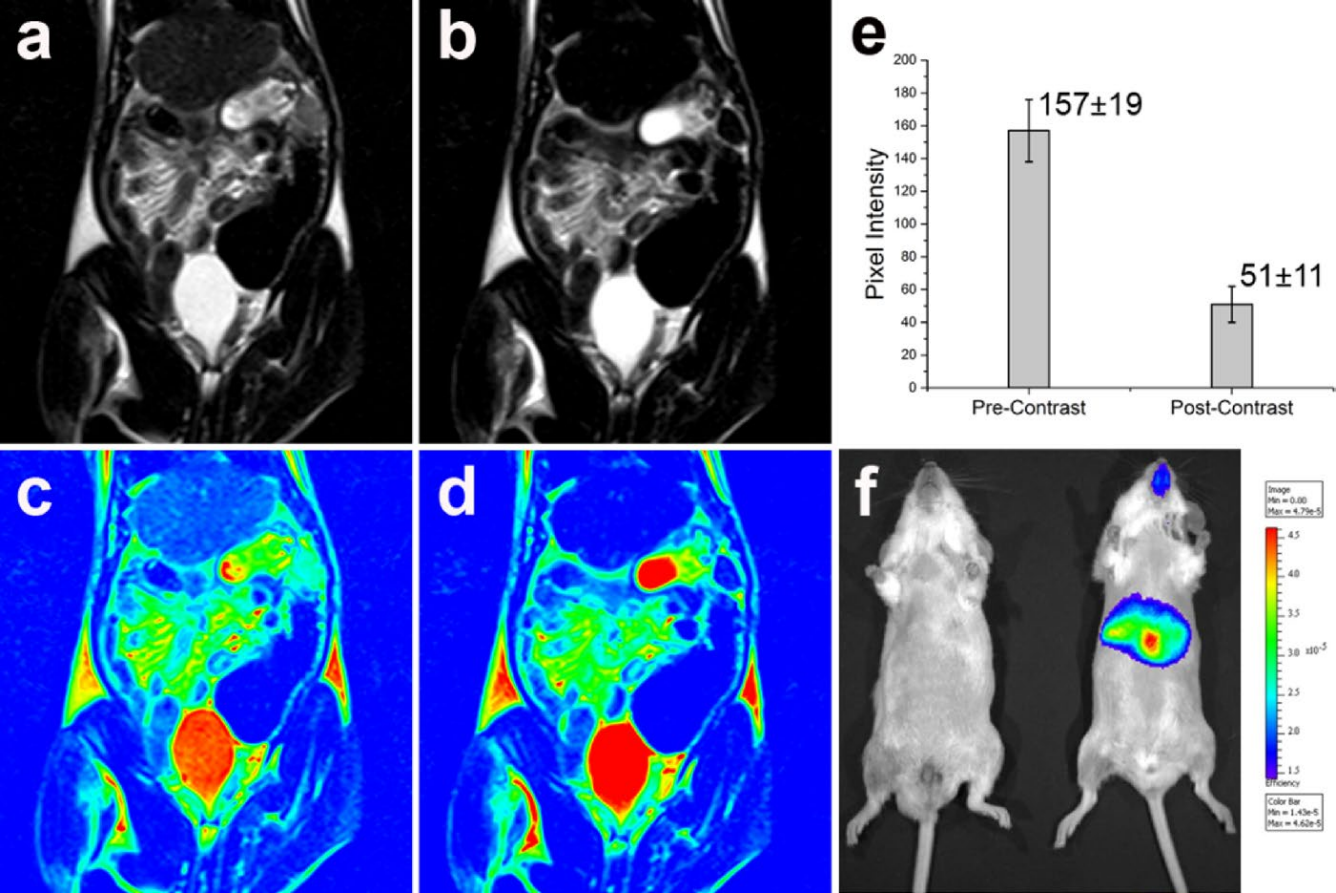
In vivo evaluation of P-SPIONs. Pre and post contrast in vivo MR images (**a**) and (**b**). The pseudo coloured images generated from (**a**) and (**b**) for easy identification are shown in (**c**) and (**d**). (**e**) The percentage signal intensity variation from pre to post contrast MR image of liver fibrosis rat model administered with P-SPIONs. (**f**) In vivo optical images of control and fibrosis induced mice model administered with PSPION-AT. Images were acquired after 15 min of intravenous administration of PSPION-AT through tail vein.

Development of liver fibrosis in CCl_4_ induced rat model was confirmed by the LFT results of the blood samples. Elevated level of liver enzymes, AST (1809 ± 469 units/l) and ALT (1446 ± 531 units/l) in comparison with control animals (AST—194 ± 121 units/l and ALT—59.5 ± 3.5 units/l) indicate the development of liver fibrosis. MRI of the anesthetized animals was performed after 15 min of tail vein injection of P-SPIONs. Post contrast T_2_ weighted images of liver showed significant hypo-intensity with a reduction in the signal intensity (SI) (Fig. 4a–e). The signal reduction indicates cumulative iron uptake by Kupffer’s cells. The average SI value decreases from 157 ± 19 (pre-contrast images) to 51 ± 11 (post contrast images) with 67% variation providing a remarkable negative contrast effect (Fig. 4c).

On detailed inspection of the MR image, the presence of few streaky linear hyper intense areas is visualized in the hypo intense liver, indicating the fibrotic regions in the affected liver. The pre to post contrast signal intensity ratio of P-SPIONs is very high (67%) compared to the 39% and 55% of C-USPIONs and D-SPIONs reported earlier (Fig. S7). The difference in pre to post contrast signal intensity ratio is statistically significant (*p* < 0.05) between all the three groups considered. This difference can be attributed to the effect of ASGPR mediated endocytosis in liver due to the presence of pullulan^15,16^. ASGPR is present in large numbers on the sinusoidal cell membrane of hepatocytes and mediates the endocytosis of desialylated glycoproteins containing terminal galactose or N-acetylgalactosamine^40^. ASGPR-mediated liver targeted delivery of Dox-loaded galactosamine-conjugated albumin nanoparticles and lactoferrinconjugated PEGylated liposomes have been well established^41,42^. Rekha et al. have used a conjugate of polyethyleneimine and pullulan for liver cell gene delivery^43^. The high affinity of pullulan to ASGPR has also been confirmed in silico through molecular docking^44,45^. It is reported that although other receptors on hepatocytes such as the retinoic acid receptor, bile receptor, hyaluronan receptor, low-density lipoprotein and high-density lipoprotein receptor may be targeted, ASGPR, hepatic lectin, a protein that has affinity towards oligosaccharide chains and hence, is an ideal target for hepatocyte-specific delivery. It is expressed in abundance on hepatocytes (500,000 ASGPR/hepatocyte) and minimally present elsewhere in the body, making it the most suitable targeting ligand for hepatocytes related studies^46^.

### In vivo optical imaging

Mice, confirmed with liver fibrosis as per the LFT results, with liver specific enzymes AST (318.5 ± 40) and ALT (107.6 ± 6) were selected for imaging. In vivo imaging of PSPION-AT was carried out after 15 min of tail vein injection. In each case, elevated fluorescence signal was observed from the liver of the fibrosis induced mice compared to the control ones. Organs of each mouse were excised after 1 h of administration of probe for assessing bio distribution of the nanoprobes. The liver expressed high fluorescence intensity clearly indicating ASGPR mediated targeting of P-SPIONs to the liver (Fig. 4f). Lower intensity of signal in other organs suggests lower affinity of the probe to organs other than liver, leading to organ specific imaging (Fig. S8). By integrating dual imaging efficacy, PSPION-AT could be used for optical imaging as well. NIR emitting probe avoids auto fluorescence from mouse tissues and acquired images can be visualized without the application of aggressive post-processing techniques^47–49^. The significant increase in fluorescence signal in the fibrosed liver is attributed due to the targeting of the PSPION-AT in the liver due to over-expression of ASGPR. It also implies that the conjugation of the fluorescent dye has not compromised the endocytosis of de-sialylated glycoproteins and hence, is useful in the diagnosis of the progression of liver abnormalities. Overall, the developed P-SPIONs might serve as an excellent hepatocyte specific MR contrast agent and PSPION-AT as a liver targeted fluorescent probe for the early diagnosis of liver abnormalities.

### Histopathology

**Figure 5:**
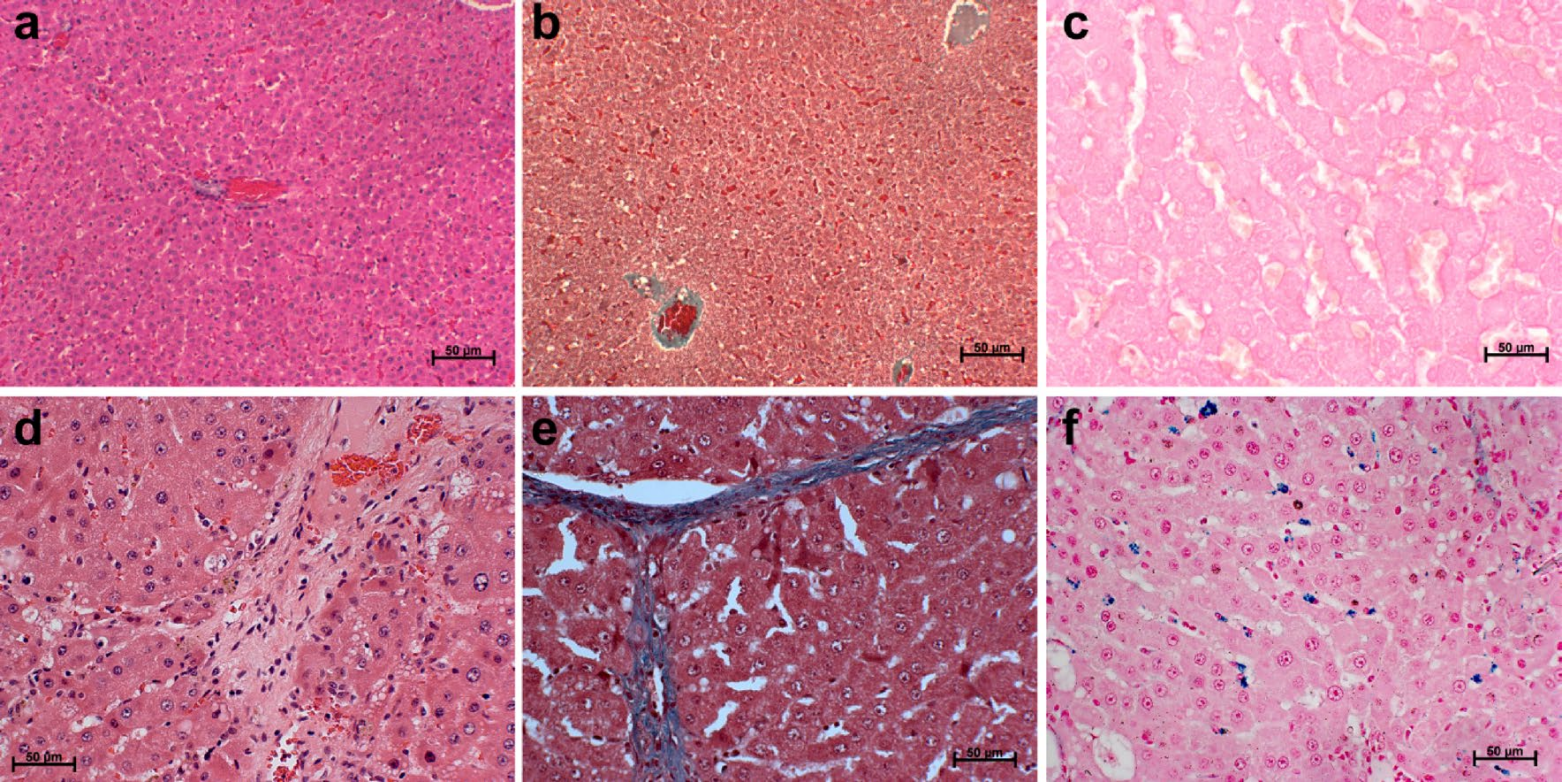
Histological evaluation of liver from normal and fibrosis model. H&E sections of (**a**) normal liver showing lobular architecture with central vein and radiating hepatic cords, (**d**) fibrosed liver showing disruption of the tissue architecture, pseudo-lobe separation with extension of fibres. MT staining of fibrosed liver (**e**) shows the presence of blue coloured fibrous accumulation that is not visible in normal liver section (**b**). Presence of iron in the P-SPION administered fibrosed liver (**f**) clearly visible in the PB stained sections whereas it is absent in the case of normal liver (**c**).

On sacrificing the animals, normal and fibrosed liver sections were evaluated pathologically using standard staining techniques like H&E, MT and PB, for routine pathological, collagen based and iron particle based evaluations, respectively. H&E and MT stained images of normal liver revealed normal liver lobular architecture with central vein and radiating hepatic cords (Fig. 5a,b). Likewise, the fibrotic liver revealed pronounced morphological alterations evidenced by disruption of the tissue architecture, moderate to severe necrosis of hepatocytes with infiltration of mononuclear cells and accumulation of fibers in perilobular and portal triad areas (Fig. 5d). The excessive accumulation of collagen fibers were very well distinguished in the MT stained section images (Fig. 5e).Variation in collagen proportionate area between control and fibrosis liver tissues calculated using the MT stained sections showed significant increase in the fibrosed tissue (Fig. S9). PB stained images showed the presence of iron in the fibrotic liver sections (Fig. 5f) compared to normal liver (Fig. 5c).

## Conclusions

In summary, a new iron oxide based hepatocyte specific MR contrast agent, P-SPION was successfully synthesized and the in vivo MR imaging of liver fibrosis was successfully evaluated. P-SPIONs provided enhanced contrast of liver parenchyma with discriminative contrast of fibrosed areas in the early stage of liver abnormalities. In vitro studies revealed the hyperthermic effect of the P-SPIONs by the application of varying magnetic field there by proving the feasibility for therapeutic application. The potential of pullulan stabilization for liver targeting via ASGPR was well proven on comparison with the SPIONs stabilized with citrate and dextran. Considering all the factors concerned with early detection of liver diseases, P-SPIONs can be considered as a new platform for in vivo liver imaging. The multimodal imaging efficiency of the probe integrating with NIR emitting Atto dye also revealed the immense scope of the probe in medical theranostics. Taking into account of the hepatocyte specific property of pullulan, the iron oxide nanoparticles can be further modified, with focus on clinical applications.

## Supplementary Information

Supplementary Information.
