# Supplementary material for: Asialoglycoprotein receptor targeted optical and magnetic resonance imaging and therapy of liver fibrosis using pullulan stabilized multi-functional iron oxide nanoprobe
Source: Sci Rep. 2021 Sep 15;11:18324. doi: 10.1038/s41598-021-97808-0 (PMC8443657; doi:10.1038/s41598-021-97808-0)
Supplement: Supplementary file 1 — Supplementary Information. [file 41598_2021_97808_MOESM1_ESM.docx]

**Supplementary Information**

**Asialoglycoprotein Receptor Targeted Optical and Magnetic Resonance Imaging and Therapy of Liver Fibrosis Using Pullulan Stabilized Multi-functional Iron Oxide Nanoprobe**

*Ariya Saraswathy^1, 2#^,ShaijuS.Nazeer^1, 3#^, Nirmala Nimi^1^, Hema Santhakumar ^1^, Parvathy Radhakrishnapillai Suma^1^,Kunnumpurathu Jibin^1^, Marina Victor^1^, Francis Boniface Fernandez^4^,Sabareeswaran Arumugam^5^, Sachin J Shenoy^6^,P.R. Harikrishna Varma^4^, Ramapurath S. Jayasree*^1^*

^1^Division of Biophotonics and Imaging,

Biomedical Technology Wing, Sree ChitraTirunal Institute for Medical Sciences &Technology, Poojappura, Thiruvananthapuram - 695 012, Kerala, India.

^2^Department of Physics, HHMSPBNSS College, Thiruvananthapuram-695 040, Kerala, India.

^3^ Department of Chemistry, Indian Institute of Space Sciences and Technology, Thiruvananthapuram - 695547, Kerala, India.

^4^Division of Bioceramics Laboratory,

^5^Division of Implant Biology

^6^Divisionof  *In Vivo* Models and Testing,

Biomedical Technology Wing, Sree Chitra Tirunal Institute for Medical Sciences &Technology, Poojappura, Thiruvananthapuram - 695 012, Kerala, India.

^#^Contributed equally to this work

*Correspondence to: [jayasree@sctimst.ac.in](mailto:jayasree@sctimst.ac.in), [jayashreemenon@gmail.com](mailto:jayashreemenon@gmail.com)

**Figure S1**. PSPION-AT was prepared by incorporating NIR emitting dye, Atto 700 with P-SPIONs through electrostatic interaction of the hydroxyl groups of SPIONs and activated COO- group of Atto 700 dye. a) The UV-Vis spectra of PSPION-AT showed an absorption peak around 700 nm. b) The fluorescence spectra of and PSPIONs-AT showed an emission maxima at 712 nm. c) The excitation - emission – efficiency contour plots for the corresponding excitation – emission imaging.

**Figure S2.** Fluorescence image and corresponding spectra of DSPION-AT with varying concentration excited at 675 nm with emission at 710 nm.


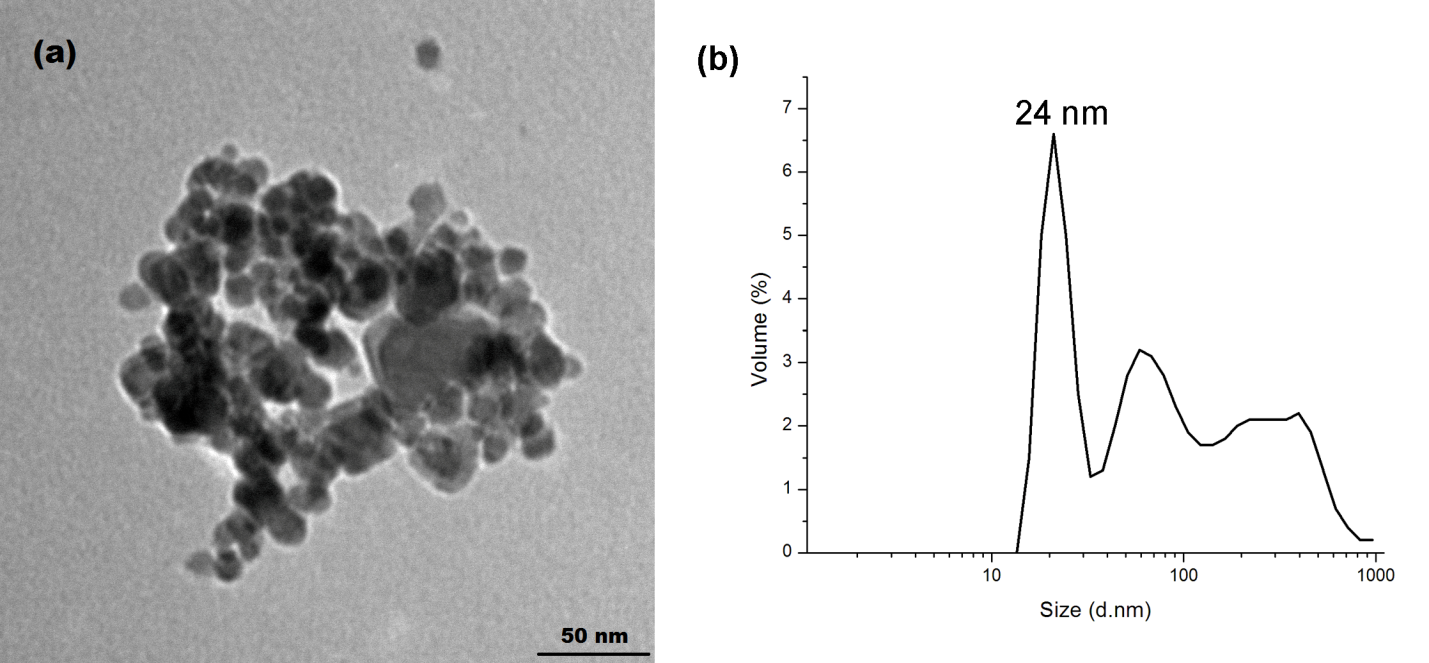
**Figure S3**. TEM image (a) and DLS graph (b) of SPIONs [Reproduced with permission from Saraswathy et al, 2014 [^1^](#_ENREF_1)]


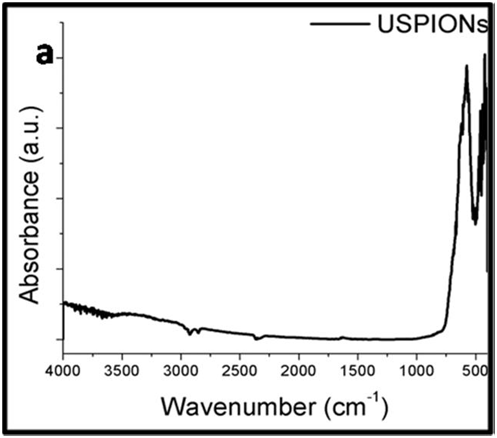


**Figure S4.** FTIR spectrum of bare SPION

**Figure S5.** Blood aggregation studies: RBCs (a-c), WBC‘s (d-f) and platelets (g-i) incubated with P-SPIONs, PEI as positive control and saline as negative control.

**Figure S6.** Cellular uptake of P-SPIONs in HepG2 cells

**Figure S7**. MRI Signal intensity comparison of P-SPION with Citrate and Dextran coated SPIONs

**Treated**

**Control**

**

Figure S8**. *Ex vivo* fluorescent image of major organs of control and P-SPION-AT treated mice.

**Figure S9**. Variation in collagen proportionate area between control and fibrosis liver tissues obtained from histological images.

**Table S1**. Specific loss power values obtained for SPIONs and P-SPIONs for varying current and magnetic field strength.

| **Sl no** | **Current through coil (A)** | **Magnetic field strength (mT)** | **SLP** | |
| --- | --- | --- | --- | --- |
|  |  |  | **SPION** | **P-SPION** |
| 1 | 200 | 14.4 | 9.79 | 0 |
| 2 | 250 | 19.33 | 20.09 | 3.60 |
| 3 | 300 | 24.166 | 29.04 | 13.81 |
| 4 | 350 | 28.99 | 40.18 | 18.99 |
| 5 | 400 | 33.83 | 50.64 | 27.62 |

Reference

1 Saraswathy, A. *et al.* Citrate coated iron oxide nanoparticles with enhanced relaxivity for in vivo magnetic resonance imaging of liver fibrosis. *Colloids and Surfaces B: Biointerfaces* **117**, 216-224, doi:https://doi.org/10.1016/j.colsurfb.2014.02.034 (2014).
